# Supplementary material for: Unraveling genomic features and phylogenomics through the analysis of three Mexican endemic Myotis genomes
Source: PeerJ. 2024 Jul 8;12:e17651. doi: 10.7717/peerj.17651 (PMC11238727; doi:10.7717/peerj.17651)
Supplement: Supplemental Information 1 [file peerj-12-17651-s001.pdf]

Unraveling genomic features and phylogenomics through the analysis of three Mexican endemic *Myotis* genomes

Edgar G. Gutiérrez[1], Jesús E. Maldonado[2], Gabriela Castellanos-Morales[3], Luis E. Eguiarte[4], Norberto Martínez-Méndez[1] and Jorge Ortega[1]*

[1]Laboratorio de Bioconservación y Manejo, Posgrado en Ciencias Químicobiológicas, Departamento de Zoología, Escuela Nacional de Ciencias Biológicas, Instituto Politécnico Nacional, Ciudad de México, Mexico.

[2]Center for Conservation Genomics, Smithsonian's National Zoo and Conservation Biology Institute, Washington, DC, United States of America.

[3]Departamento de Conservación de la Biodiversidad, El Colegio de la Frontera Sur, Unidad Villahermosa (ECOSUR-Villahermosa), Villahermosa, Tabasco, Mexico

[4]Departamento de Ecología Evolutiva, Instituto de Ecología, Universidad Nacional Autónoma de México, Ciudad de México, Mexico.

Corresponding author:

Jorge Ortega

E-mail: artibeus2@aol.com

A)

Basic Statistics

Measure	Value
Filename	allreads_Mv_1.fq.gz
File type	Conventional base calls
Encoding	Sanger / Illumina 1.9
Total Sequences	216183530
Sequences flagged as poor quality	0
Sequence length	150
%GC	42

Basic Statistics

Measure	Value
Filename	allreads_Mv_2.fq.gz
File type	Conventional base calls
Encoding	Sanger / Illumina 1.9
Total Sequences	216183530
Sequences flagged as poor quality	0
Sequence length	150
%GC	43

Per base sequence quality

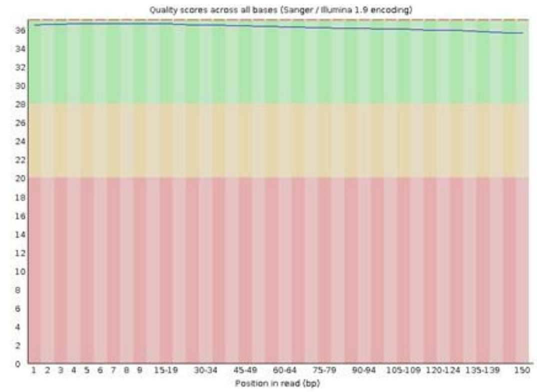

Per base sequence quality

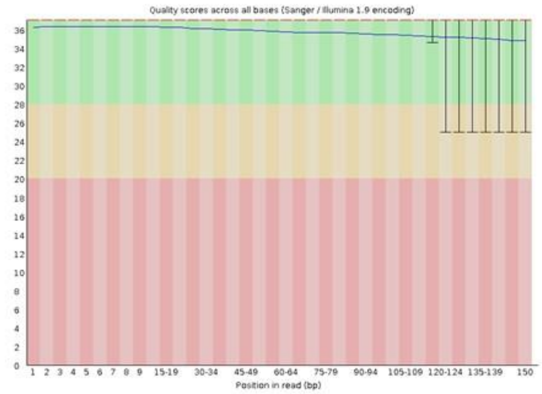

B)

Basic Statistics

Measure	Value
Filename	allreadsMplan_1.fq.gz
File type	Conventional base calls
Encoding	Sanger / Illumina 1.9
Total Sequences	232122197
Sequences flagged as poor quality	0
Sequence length	150
%GC	38

Basic Statistics

Measure	Value
Filename	allreadsMplan_2.fq.gz
File type	Conventional base calls
Encoding	Sanger / Illumina 1.9
Total Sequences	232122197
Sequences flagged as poor quality	0
Sequence length	150
%GC	38

Per base sequence quality

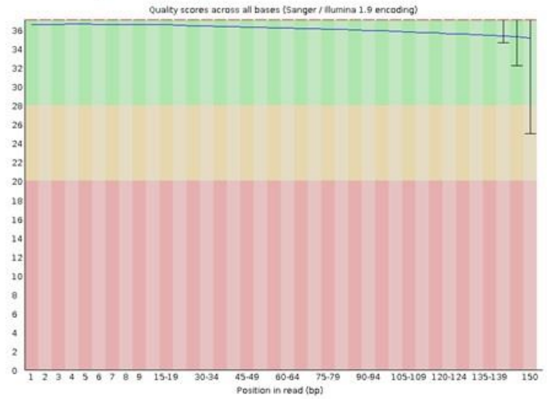

Per base sequence quality

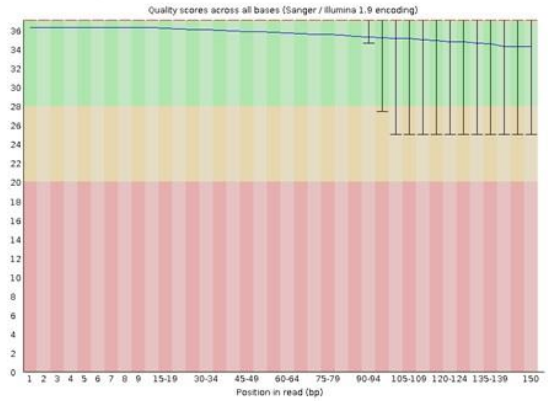

C)

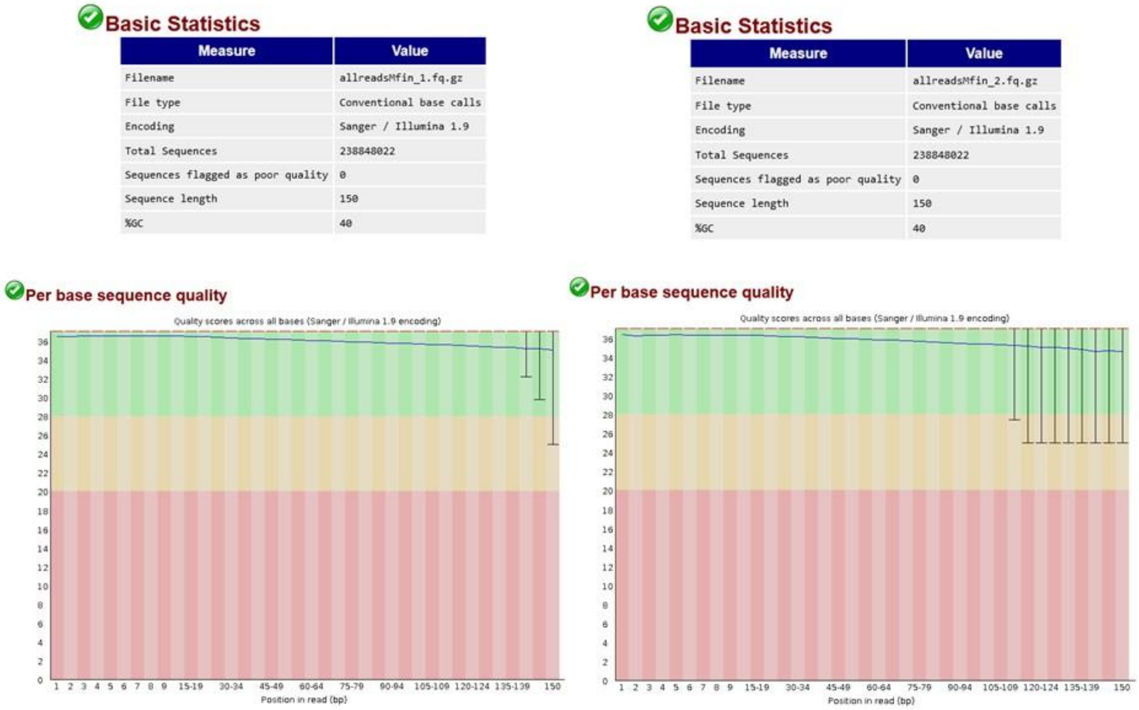

Figure S1. Partial FastQC analysis of each raw reads file. The total number of reads generated and the phred quality plot of each of the three Illumina Novaseq-6000 sequencings are shown. Files of reads in both senses (left = 5' - 3'; right= 3' - 5') of A) *M. vivesi*, B) *M. planiceps*, and C) *M. findleyi* are shown.

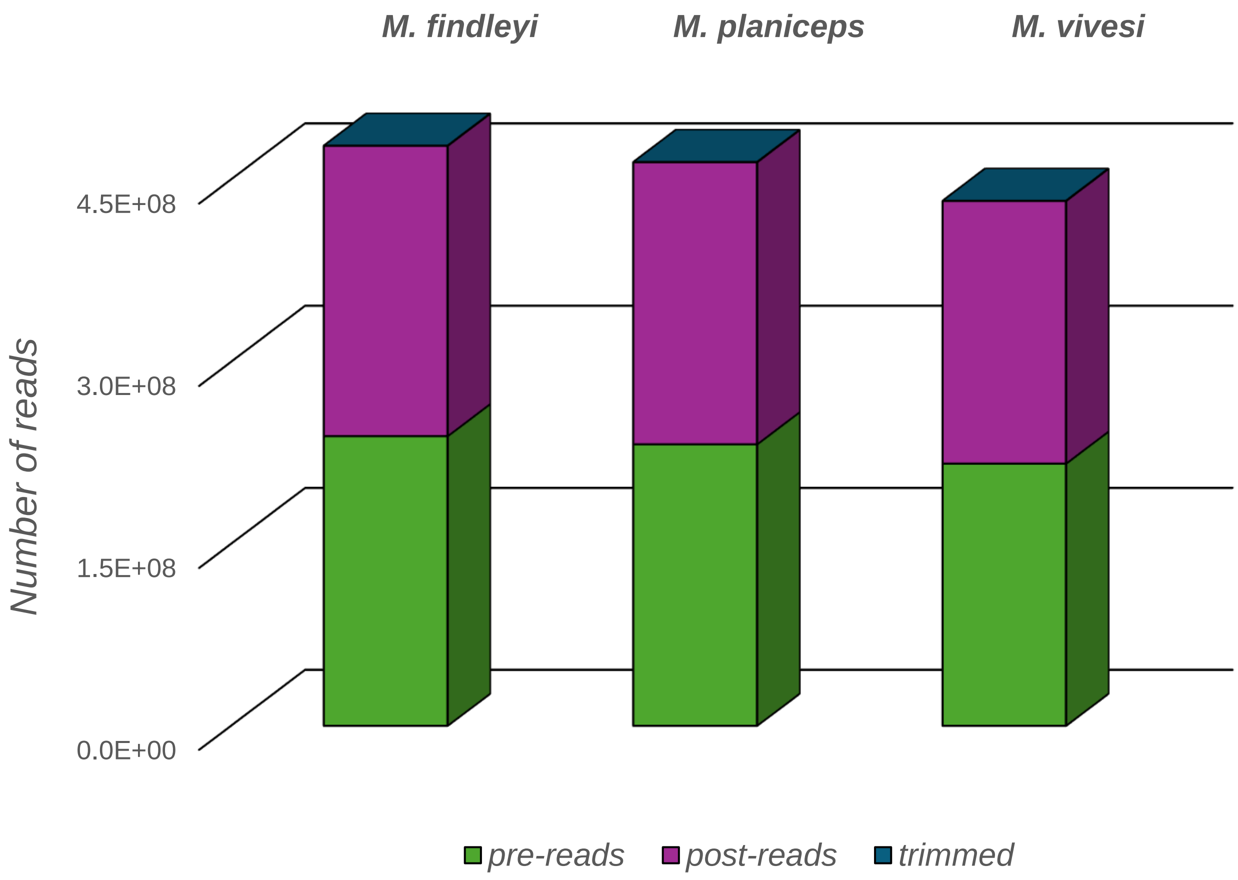

Figure S2. **Number of reads pre- and post-trimming in each reads file for the three *Myotis* species analyzed here.**

A)

Basic Statistics

Measure	Value
Filename	allreads_Mv_1.paired.fq.gz
File type	Conventional base calls
Encoding	Sanger / Illumina 1.9
Total Sequences	216175730
Sequences flagged as poor quality	0
Sequence length	36-150
%GC	42

Basic Statistics

Measure	Value
Filename	allreads_Mv_2.paired.fq.gz
File type	Conventional base calls
Encoding	Sanger / Illumina 1.9
Total Sequences	216175730
Sequences flagged as poor quality	0
Sequence length	36-150
%GC	42

Per base sequence quality

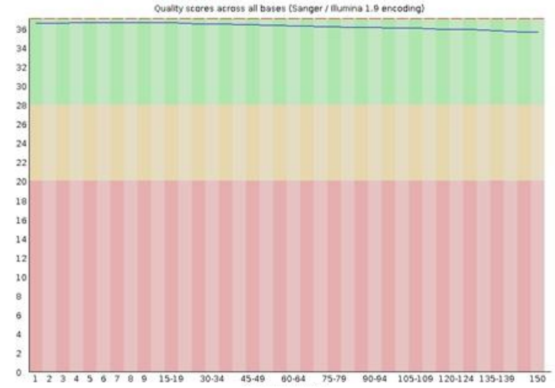

Per base sequence quality

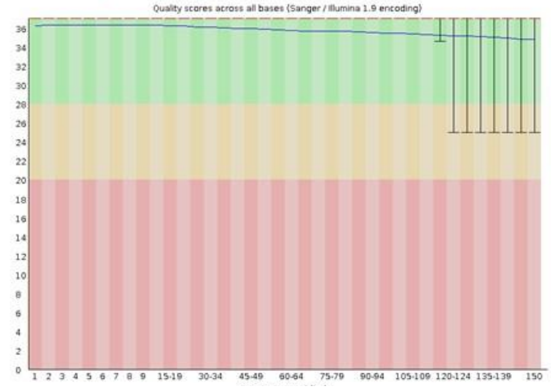

B)

Basic Statistics

Measure	Value
Filename	allreadsMplan_1.paired.fq.gz
File type	Conventional base calls
Encoding	Sanger / Illumina 1.9
Total Sequences	232110228
Sequences flagged as poor quality	0
Sequence length	36-150
%GC	38

Basic Statistics

Measure	Value
Filename	allreadsMplan_2.paired.fq.gz
File type	Conventional base calls
Encoding	Sanger / Illumina 1.9
Total Sequences	232110228
Sequences flagged as poor quality	0
Sequence length	36-150
%GC	38

Per base sequence quality

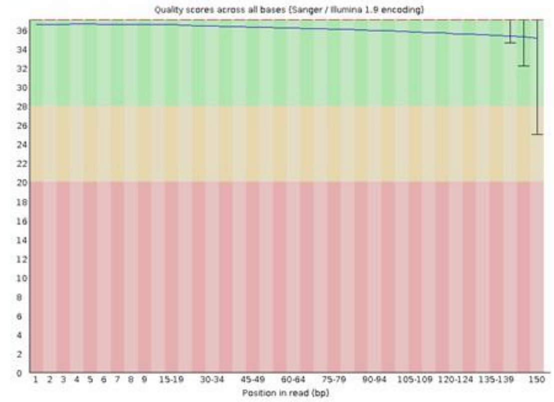

Per base sequence quality

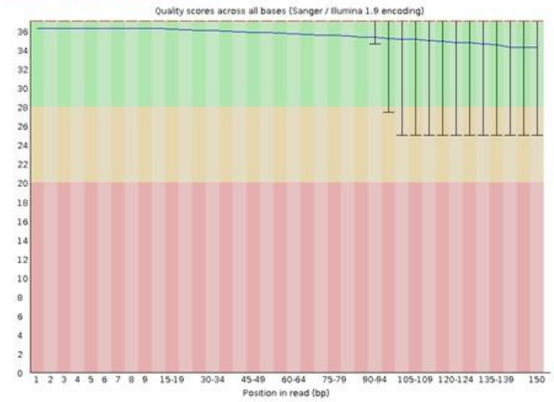

C)

Basic Statistics

Measure	Value
Filename	allreadsMfin_1.paired.fq.gz
File type	Conventional base calls
Encoding	Sanger / Illumina 1.9
Total Sequences	238840302
Sequences flagged as poor quality	0
Sequence length	36-150
%GC	40

Basic Statistics

Measure	Value
Filename	allreadsMfin_2.paired.fq.gz
File type	Conventional base calls
Encoding	Sanger / Illumina 1.9
Total Sequences	238840302
Sequences flagged as poor quality	0
Sequence length	36-150
%GC	40

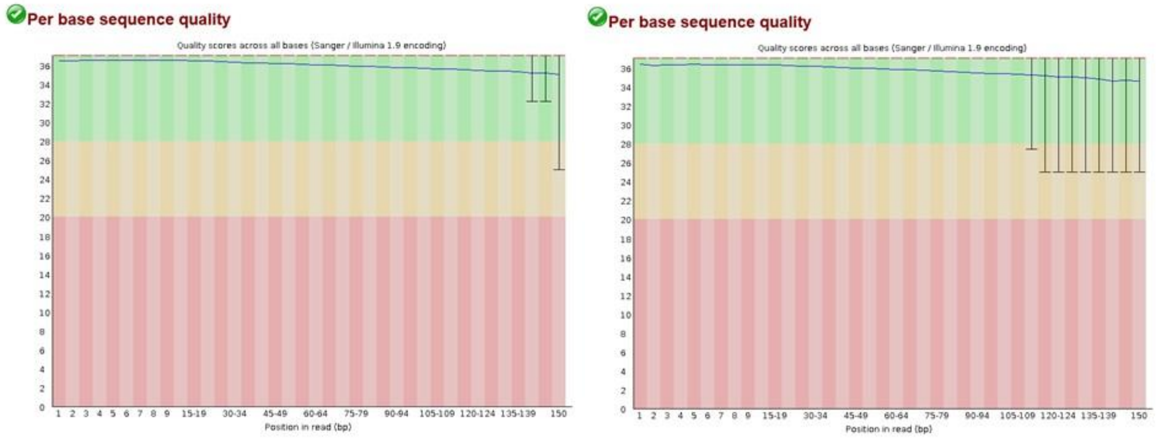

Figure S3. Partial FastQC analysis of each post- trimming reads file. The total number of reads trimmed and the phred quality plot are shown. Files of post-trimming reads in both senses (left = 5' - 3'; right= 3' - 5') of A) *M. vivesi*, B) *M. planiceps*, and C) *M. findleyi* are shown.

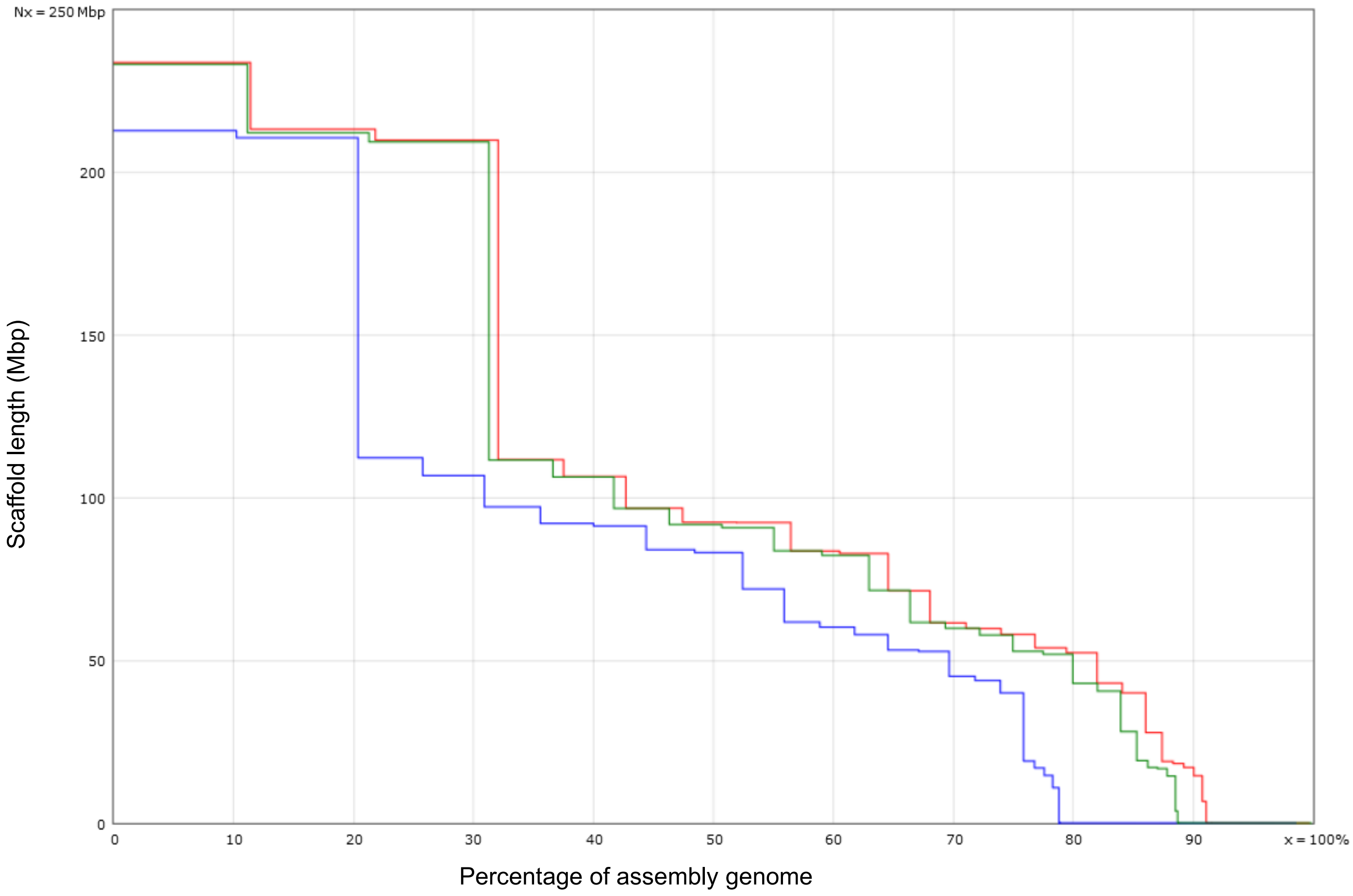

Figure S4. The NGx plot provides information on the size of the scaffolds as a percentage of the total length of the genome assembly. The red, green, and blue lines represent the scaffold length of the genome assembly of *M. findleyi*, *M. vivesi*, and *M. planiceps*, respectively.

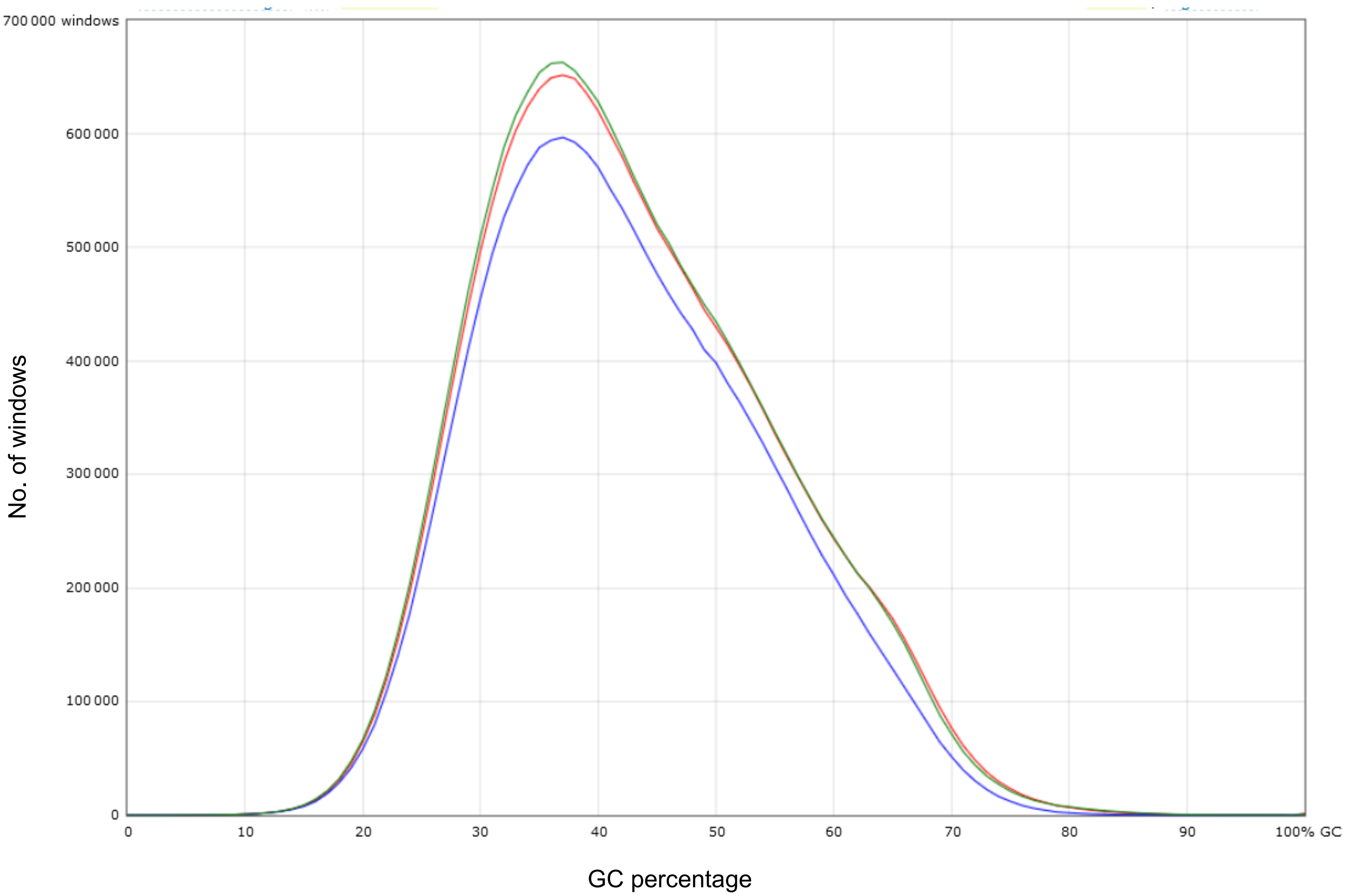

Figure S5. The GC content of the three Mexican endemic Myotis species. Scaffolds are broken into nonoverlapping 100 bp windows. Plot shows number of windows for each GC percentage. Red, blue, and green lines represent the GC content of *M. findleyi*, *M. planiceps*, and *M. vivesi*, respectively.

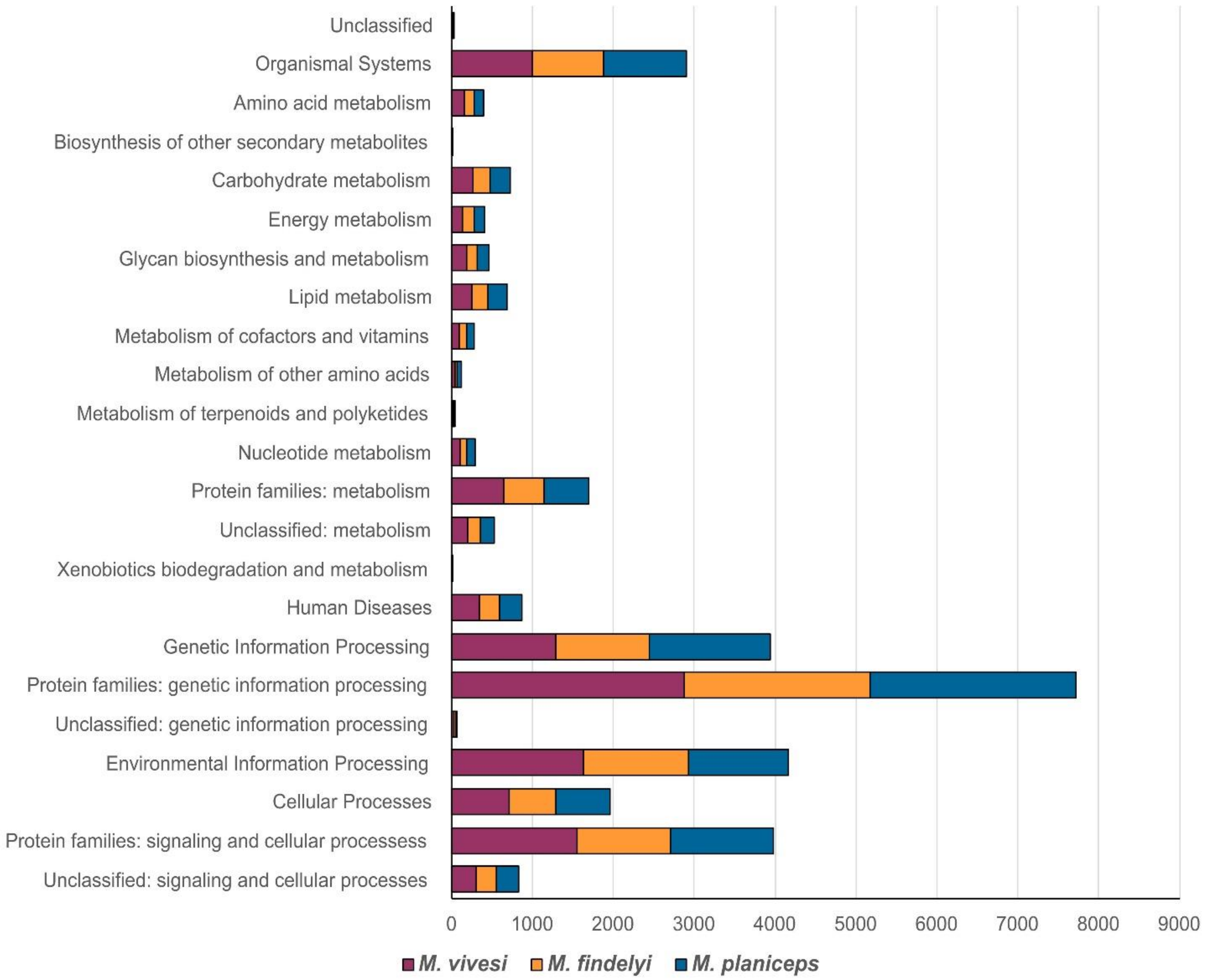

Figure S6. Kyoto Encyclopedia of Genes and Genomes (KEGG) pathways of differentially expressed genes. Total amount of annotated genes (*X-axis*) versus KEGG categories (*Y-axis*).

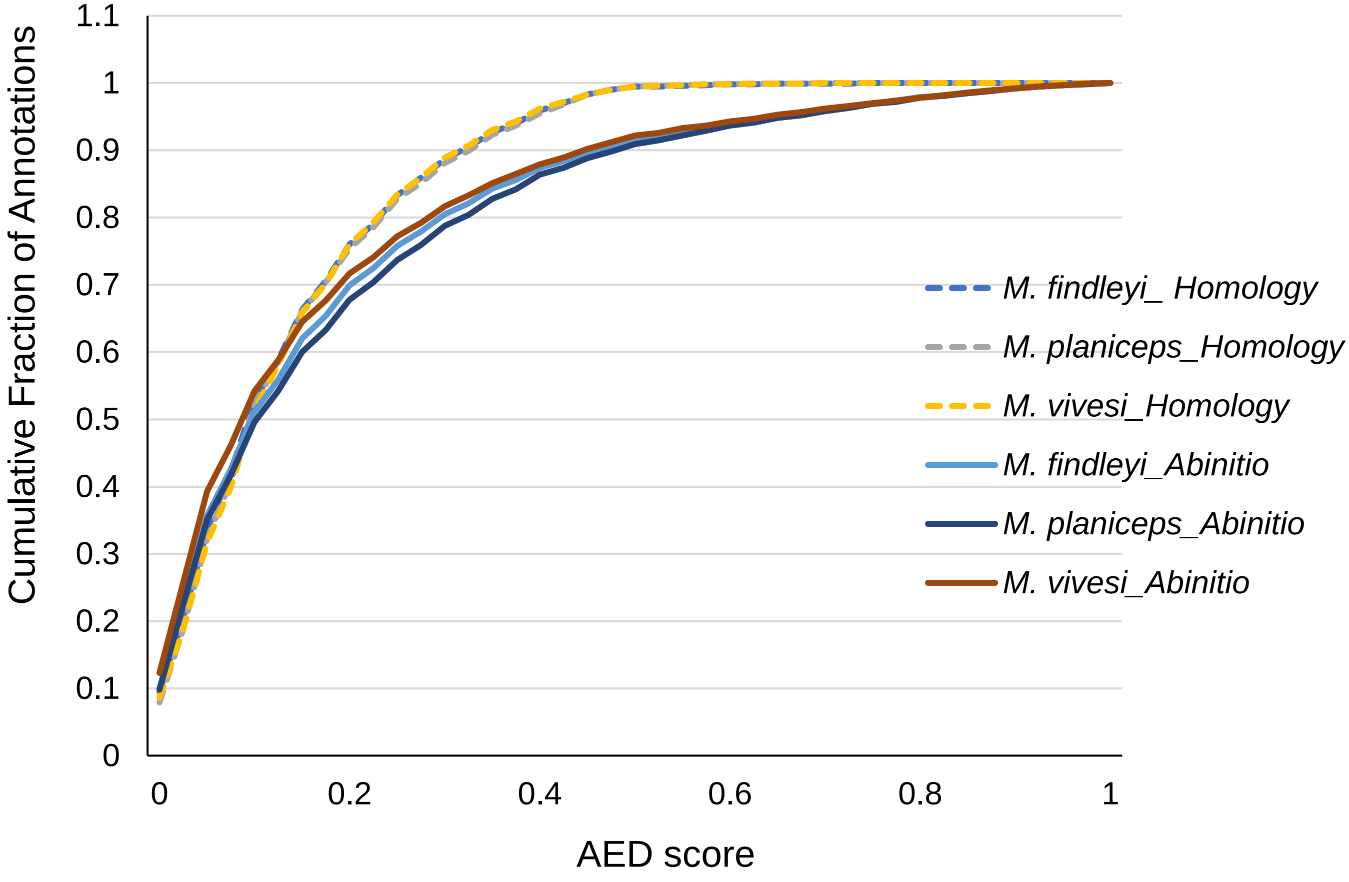

Figure S7. The cumulative fraction of annotation edit distance (AED) distribution. Evidence the quality of the annotation in both gene prediction approaches of the three *Myotis* genomes sequences.

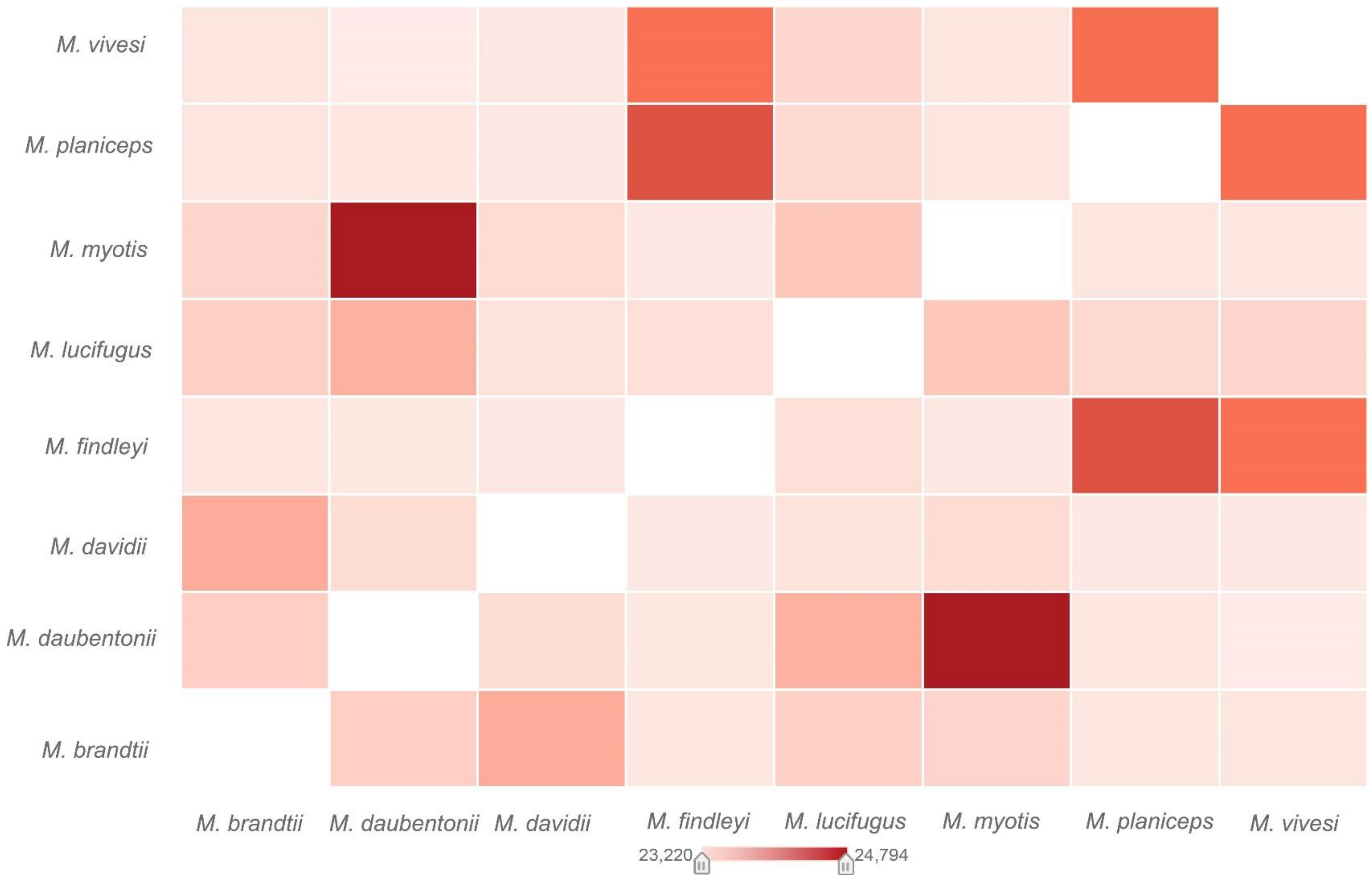

Figure S8. Heat map of orthologous genes shared between *Myotis* species. The number of genes with homology between *Myotis* species is shown, where the color intensity reflects a greater number of shared genes and vice versa.
