# Supplementary material for: Unraveling genomic features and phylogenomics through the analysis of three Mexican endemic Myotis genomes
Source: PeerJ. 2024 Jul 8;12:e17651. doi: 10.7717/peerj.17651 (PMC11238727; doi:10.7717/peerj.17651)
Supplement: Supplemental Information 3 [file peerj-12-17651-s003.docx]

**Unraveling genomic features and phylogenomics through the analysis of three Mexican endemic *Myotis* genomes**

Edgar G. Gutiérrez^1^, Jesús E. Maldonado^2^, Gabriela Castellanos-Morales^3^, Luis E. Eguiarte^4^, Norberto Martínez-Méndez^1^ and Jorge Ortega^1^*

^1^Laboratorio de Bioconservación y Manejo, Posgrado en Ciencias Químicobiológicas, Departamento de Zoología, Escuela Nacional de Ciencias Biológicas, Instituto Politécnico Nacional, Ciudad de México, Mexico.

^2^Center for Conservation Genomics, Smithsonian’s National Zoo and Conservation Biology Institute, Washington, DC, United States of America.

^3^Departamento de Conservación de la Biodiversidad, El Colegio de la Frontera Sur, Unidad Villahermosa (ECOSUR-Villahermosa), Villahermosa, Tabasco, Mexico

^4^Departamento de Ecología Evolutiva, Instituto de Ecología, Universidad Nacional Autónoma de México, Ciudad de México, Mexico.

Corresponding author:

Jorge Ortega

E-mail: artibeus2@aol.com

| **Adapter** | **Sequence** |
| --- | --- |
| > 5'_Adapter: | AGATCGGAAGAGCGTCGTGTAGGGAAAGAGTGTAGATCTCGGTGGTCGCCGTATCATT |
| >3'_Adapter: | GATCGGAAGAGCACACGTCTGAACTCCAGTCACGGATGACTATCTCGTATGCCGTCTTCTGCTTG |

**Table S1.** **Adapter sequences used in sequencing through the Illumina Novaseq 6000 platform.**

**Table S2. Assembly statistics resulting from the genome assemblies obtained with de novo approach in MaSuRCA of each *Myotis* species analyzed.**

| **Statistics** | ***M. vivesi*** | ***M. planiceps*** | ***M. findleyi*** |
| --- | --- | --- | --- |
| **# scaffolds** | 149,140 | 456,935 | 155,779 |
| **# scaffolds (>= 1000 bp)** | 136,416 | 354,774 | 139,268 |
| **# scaffolds (>= 5000 bp)** | 96,357 | 118,977 | 91,978 |
| **# scaffolds (>= 10000 bp)** | 64,916 | 43,736 | 60,766 |
| **# scaffolds (>= 25000 bp)** | 22,099 | 3,630 | 21,788 |
| **# scaffolds (>= 50000 bp)** | 4,473 | 105 | 5,257 |
| **Largest contig (bp)** | 189,978 | 106,939 | 224,674 |
| **Total length (Gbp)** | 1.94 | 1.87 | 1.93 |
| **N50 (Mb)** | 23,188 | 7,378 | 24,338 |
| **N90 (Mb)** | 6,686 | 1,777 | 6,220 |
| **L50** | 25,033 | 72,144 | 22,745 |
| **L90** | 84,037 | 270,266 | 82,635 |
| **GC (%)** | 42.70 | 41.98 | 42.79 |

**Table S4.** **Assembly statistics resulting from the genome assemblies obtained with MaSuRCA, after scaffolding and gap-filling in each of the *Myotis* species analyzed.**

| **Statistics** | ***M. vivesi*** | ***M. planiceps*** | ***M. findleyi*** |
| --- | --- | --- | --- |
| **# scaffolds** | 58,658 | 256,303 | 48,382 |
| **# scaffolds (>= 1000 bp)** | 44,985 | 154,381 | 30,889 |
| **# scaffolds (>= 5000 bp)** | 12,071 | 10,156 | 7,642 |
| **# scaffolds (>= 10000 bp)** | 5,024 | 1,171 | 4,288 |
| **# scaffolds (>= 25000 bp)** | 1,010 | 46 | 1,266 |
| **# scaffolds (>= 50000 bp)** | 142 | 29 | 280 |
| **Largest scaffold (Mb)** | 233.3 | 212.9 | 233.9 |
| **Total length (Gb)** | 2.06 | 2.08 | 2.05 |
| **N50 (Mb)** | 91.8 | 83.2 | 92.5 |
| **N90 (Mb)** | 29,765 | 2,125 | 17,114,864 |
| **L50** | 7 | 9 | 7 |
| **L90** | 682 | 61,948 | 22 |
| **GC (%)** | 42.72 | 42.50 | 42.91 |

**Table S5.** **Number of predicted genes in the three genomes of Mexican endemic *Myotis* species functionally annotated using indicated databases (column 1).**

|  | ***M. findleyi*** | ***M. vivesi*** | ***M. planiceps*** |
| --- | --- | --- | --- |
| **Genes** | 21,394 | 23,851 | 23,444 |
| **InterProScan** | 18,439 | 22,351 | 22,229 |
| **UniProt/Swiss-Prot** | 19,663 | 21,754 | 21,327 |
| **KEGG** | 9,546 | 11,868 | 10,719 |

**Table S6. Statistics of the result of grouping of orthologous genes with Orthofinder, using a total of six species of the genus *Myotis*.** Three species correspond to the genomes of Mexican endemic *Myotis* and another three to *Myotis* available in the NCBI database.

| Number of species | 8 |
| --- | --- |
| Number of genes | 179,639 |
| Number of genes in orthogroups | 170,943 |
| Number of unassigned genes | 8,696 |
| Percentage of genes in orthogroups | 95.2% |
| Percentage of unassigned genes | 4.8% |
| Number of orthogroups | 20,820 |
| Number of species-specific orthogroups | 324 |
| Percentage of genes in species-specific orthogroups | 0.5% |
| Mean orthogroup size | 8.2 |
| Median orthogroup size | 8 |
| G50 (assigned genes) | 8 |
| G50 (all genes) | 8 |
| O50 (assigned genes) | 6,197 |
| O50 (all genes) | 6,740 |
| Number of orthogroups with all species present | 9,398 |
| Number of single-copy orthogroups | 4,789 |
